# Drug-induced alterations in tumour perfusion yield increases in tumour cell radiosensitivity

**DOI:** 10.1054/bjoc.2001.2123

**Published:** 2001-11

**Authors:** K L Bennewith, R E Durand

**Affiliations:** Medical Biophysics Department, British Columbia Cancer Research Centre, 601 West 10th Avenue, Vancouver, V5Z 1L3, Canada

**Keywords:** hypoxia, tumour perfusion, radiosensitivity, pentoxifylline, diltiazem

## Abstract

The perfusion of human tumour xenografts was manipulated by administration of diltiazem and pentoxifylline, and the extent that observed changes in tumour perfusion altered tumour radiosensitivity was determined. 2 tumour systems having intrinsically different types of hypoxia were studied. The responses of SiHa tumours, which have essentially no transient hypoxia, were compared to the responses of WiDr tumours, which contain chronically and transiently hypoxic cells. We found that relatively modest increases in net tumour perfusion increased tumour cell radiosensitivity in WiDr tumours to a greater extent than in SiHa tumours. Moreover, redistribution of blood flow within WiDr tumours was observed on a micro-regional level that was largely independent of changes in net tumour perfusion. Through fluorescence-activated cell sorting coupled with an in vivo–in vitro cloning assay, increases in the radiosensitivity of WiDr tumour cells at intermediate levels of oxygenation were observed, consistent with the expectation that a redistribution of tumour blood flow had increased oxygen delivery to transiently hypoxic tumour cells. Our data therefore suggest that drug-induced changes in tumour micro-perfusion can alter the radiosensitivity of transiently hypoxic tumour cells, and that increasing the radiosensitivity of tumour cells at intermediate levels of oxygenation is therapeutically relevant. © 2001 Cancer Research Campaign   http://www.bjcancer.com


*British Journal of Cancer* (2001) **85**(10), 1577–1584
© 2001 Cancer Research Campaign
doi: 10.1054/ bjoc.2001.2123, available online at http://www.idealibrary.com on IDEAL

http://www.bjcancer.com

### Drug-induced alterations in tumour perfusion yield increases in tumour cell radiosensitivity

**KL Bennewith and RE Durand**

Medical Biophysics Department, British Columbia Cancer Research Centre, 601 West 10th Avenue, Vancouver, B.C., Canada V5Z 1L3

**Summary** The perfusion of human tumour xenografts was manipulated by administration of diltiazem and pentoxifylline, and the extent that observed changes in tumour perfusion altered tumour radiosensitivity was determined. 2 tumour systems having intrinsically different types of hypoxia were studied. The responses of SiHa tumours, which have essentially no transient hypoxia, were compared to the responses of WiDr tumours, which contain chronically and transiently hypoxic cells. We found that relatively modest increases in net tumour perfusion increased tumour cell radiosensitivity in WiDr tumours to a greater extent than in SiHa tumours. Moreover, redistribution of blood flow within WiDr tumours was observed on a micro-regional level that was largely independent of changes in net tumour perfusion. Through fluorescence-activated cell sorting coupled with an in vivo–in vitro cloning assay, increases in the radiosensitivity of WiDr tumour cells at intermediate levels of oxygenation were observed, consistent with the expectation that a redistribution of tumour blood flow had increased oxygen delivery to transiently hypoxic tumour cells. Our data therefore suggest that drug-induced changes in tumour micro-perfusion can alter the radiosensitivity of transiently hypoxic tumour cells, and that increasing the radiosensitivity of tumour cells at intermediate levels of oxygenation is therapeutically relevant. © 2001 Cancer Research Campaign http://www.bjcancer.com

**Keywords**: hypoxia; tumour perfusion; radiosensitivity; pentoxifylline; diltiazem

Tumour cells exist at various levels of oxygenation and can therefore respond differently to a given dose of radiation. Since Thomlinson and Gray (1955) first observed cords of viable cells surrounding blood vessels in histological tumour sections, numerous studies have focused on strategies designed to target chronically hypoxic tumour cells. More recently, it has been postulated that tumour cell subpopulations at intermediate levels of oxygenation may also have a large impact on tumour response to radiation (Wouters and Brown, 1997). The possibility of targeting intermediately oxygenated cells, and the resultant effect that destroying these cells would have on overall tumour radiosensitivity, are avenues of research that need to be explored. Altering tumour perfusion to favour increased oxygen delivery to intermediately hypoxic cells prior to therapeutic irradiation may have an important impact on solid tumour response to radiation. The calcium channel blocker diltiazem and the haemorrheologic agent pentoxifylline were used as tools in order to study the effects that changes in tumour perfusion have on the radiosensitivity of various subpopulations of tumour cells.

Calcium channel blockers have the general ability to affect calcium-mediated cellular processes by blocking the uptake of calcium ions through plasma membrane receptors (Greenberg, 1987). Benzothiazepine-derived calcium channel blockers, such as diltiazem hydrochloride, act primarily to dilate the principal coronary arteries and some systemic arteries, resulting in a decrease in total peripheral resistance and systemic blood pressure (Arcuri et al, 1998a). Diltiazem has been shown to increase tumour perfusion and tumour cell radiosensitivity in SCCVII/St tumours (Wood and Hirst, 1989). Diltiazem also increased tumour perfusion and oxygenation in the Ehrlich ascites tumour model, which was correlated with tumour regression after irradiation (Muruganandham et al, 1999).

Pentoxifylline is a dimethylxanthine derived hemorrheologic agent that has been shown to increase the deformability of red blood cells and leukocytes (Ehrly, 1978; Armstrong et al, 1990; Arcuri et al, 1998b). These observations are important when considering the increased blood cell rigidity and associated increase in blood viscosity that can result from exposure of blood cells to the hypoxic conditions found in many tumours (Van Nueten and Vanhoutte, 1980; Hakim and Macek, 1988). Decreased flow through tortuous tumour vasculature can make micro-regions of the blood hypoxic as the tumour tissue utilizes the available oxygen. The concomitant decrease in micro-regional blood pH decreases the flexibility of blood cells, which further impairs blood flow through the narrow vessels. Administration of pentoxifylline can result in an increased flexibility of blood cells (with a decrease in whole blood viscosity) that can increase blood flow through narrow tumour vasculature. Pentoxifylline has been shown to decrease interstitial fluid pressure and increase net tumour perfusion, oxygenation, and net radiosensitivity in various experimental tumour systems (Lee et al, 1992, 1993, 1994; Song et al, 1992, 1994; Honess et al, 1993, 1995; Kelleher et al, 1998).

We studied diltiazem and pentoxifylline-induced alterations in the perfusion and radiosensitivity of SiHa and WiDr human tumour xenografts. Alterations in net tumour perfusion were measured using a modified $^{86}$Rb extraction method, while changes in the micro-regional distribution of tumour blood flow were studied using a dual staining mismatch technique. When combined with information from an in vivo–in vitro cloning assay including

*Received 11 December 2000*
*Revised 17 July 2001*
*Accepted 19 July 2001*

*Correspondence to:* RE Durand fluorescence-activated cell sorting (FACS) analysis, our data address the degree to which drug-induced alterations in tumour macro- and micro-perfusion can affect the radiosensitivity of tumour cell subpopulations.

### MATERIALS AND METHODS

### Mice and tumours

The tumours were derived from SiHa, a human cervical squamous cell carcinoma (Friedl et al, 1970) and WiDr, a human colon adenocarcinoma (Noguchi et al, 1979), cell lines. Both were obtained as cultured cell lines (ATCC, Rockville, Maryland), grown in SCID mice, and maintained by intramuscular transplant. Experimental tumours were grown as dorsal subcutaneous implants in NOD-SCID mice (bred in-house) for all perfusion, mismatch and sorting experiments. All procedures were performed in accordance with the ethical standards of the University of British Columbia Committee on Animal Care and the Canadian Council on Animal Care, which conform in every way to the UKCCR Guidelines (UKCCR, 1998).

### Drugs

The drugs diltiazem hydrochloride (ICN Biomedicals, Costa Mesa, California) and pentoxifylline (Sigma, Oakville, Ontario) were dissolved in PBS on the day of each experiment. The appropriate drug concentrations were delivered in an i.p. injection volume of 0.01 ml$^{-1}$ gram body weight.

### Modified $^{86}$rubidium extraction technique

The classical $^{86}$rubidium ($^{86}$Rb) extraction method is based on the observation that after a bolus injection of $^{86}$rubidium chloride ($^{86}$RbCl) into the bloodstream of an experimental animal, the uptake of the isotope by each tissue is proportional to the fraction of the cardiac output flowing through that tissue (Sapirstein, 1958). Gullino and Grantham (1961) validated the method for measuring net blood flow in implanted tumours. A primary limitation of the technique is that each blood flow determination is terminal, and therefore only a single perfusion measurement can be performed in a given animal. We developed a modified $^{86}$Rb extraction method to enable multiple tumour blood flow measurements in the same mouse. The procedure allowed one perfusion measurement as a control or baseline value without sacrifice of the animal. A blood flow-modifying agent could then be administered and a second perfusion determination performed. Each animal (and tumour) was therefore used as its own control.

$^{86}$Rubidium chloride (Amersham Pharmacia Biotech, Buckinghamshire, England) was diluted in PBS to an activity of ~ 3.7 MBq of $^{86}$Rb per 0.1 ml injection. The method involved the use of a solid-state radiation detection probe externally positioned over the implanted tumour during each of 2 sequential intravenous injections of $^{86}$RbCl (Figure 1). The animal was treated with the appropriate concentration of drug at a given time before the second $^{86}$RbCl injection. Activity from the tumour area was measured for 90 s after each isotope injection, which has previously been established as the time in which tumour uptake of $^{86}$Rb is at a plateau (Zanelli and Fowler, 1974). After a short period of time, the residual tumour activity from the first $^{86}$RbCl injection would not be representative of tumour blood flow since significant amounts of the isotope would have recirculated and redistributed throughout the animal. Therefore the activity remaining from the first $^{86}$RbCl injection was used as the background radiation signal for the second injection. The radioactivity of the tail was also measured after each injection in order to determine the amount of injection solution that remained at the injection site. Mice were excluded from analysis if the activity remaining in the tail from either $^{86}$RbCl injection was more than 10% of the injected activity.

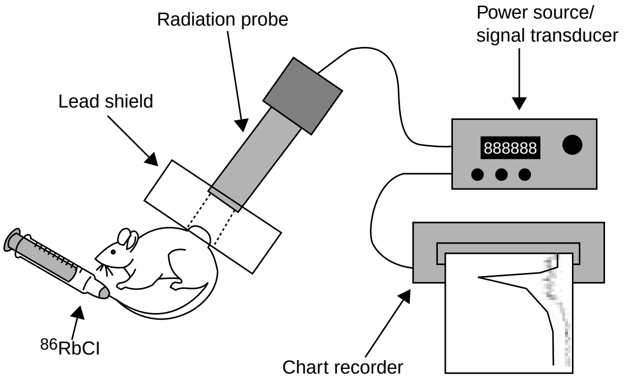

**Figure 1** Experimental apparatus for the modified $^{86}$RbCl extraction procedure. The lead shield was designed with an aperture so the radioactivity signal from the tumour area could be isolated from the overall radioactivity of the animal. The signal from the tumour area is normalized for activity from the skin overlying the tumour (see text)

Animals were killed by cervical dislocation 90 s after the second isotope injection. The tumour and skin overlying the tumour were excised, weighed and the radioactivities measured in a Cobra II AutoGamma well-type radiation counter (Packard Instrumentals, Meriden, Connecticut). The external radioactivity signal from the tumour area was normalized for the skin overlying the tumour to provide a radioactivity estimate for the tumour alone. Data are expressed in terms of the percentage of injected activity per gram of tumour tissue, which is proportional to the percentage of the cardiac output per gram of tissue (%CO g$^{-1}$).

### Dual staining mismatch

The micro-regional distribution of tumour blood flow was assessed via a dual-staining mismatch technique designed to observe transient alterations in tumour perfusion (Trotter et al, 1989a, 1989b). The fluorescent bisbenzimide dye Hoechst 33342 (0.5 mg per mouse delivered in 0.05 ml PBS) was administered by intravenous injection to a tumour-bearing mouse, followed 35 min later by i.v. injection of the carbocyanine derivative $DiOC_7$ (0.1 mg per mouse in 0.05 ml 75% DMSO). Pentoxifylline was injected intraperitoneally at 15 or 30 min before $DiOC_7$ injection. The animals were sacrificed 5 min after carbocyanine injection and the tumours were excised, embedded, frozen and sectioned. The Hoechst and carbocyanine stains were thus allowed to perfuse the tumour for at least 5 minutes before drug treatment or tumour excision respectively. This experimental design allowed the staining pattern from the first dye to be quantitatively compared with alterations in the delivery of the second dye induced by the pentoxifylline treatment (Figure 2). Representative microscopic images of the tumour sections were

 *© 2001 Cancer Research Campaign*

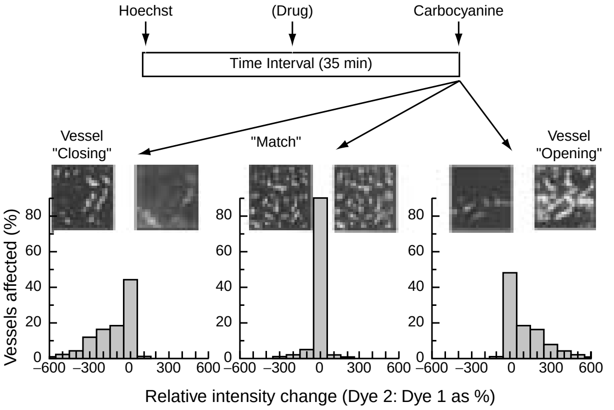

**Figure 2** Schematic for the dual-staining mismatch protocol. Tumour areas that stained more brightly with carbocyanine relative to Hoechst (i.e. positive intensity changes) were adjacent to blood vessels that 'opened' during the time interval. Tumour areas that stained less brightly with carbocyanine relative to Hoechst (i.e. negative intensity changes) were adjacent to blood vessels that 'closed' during the time interval. Tumour areas that had 'matched' staining patterns were adjacent to blood vessels that did not increase or decrease perfusion by more than 2-fold during the time between dye injections digitized and analysed by locally developed software for the fluorescent image processing system (FIPS) (Durand and LePard, 1994, 1995). 10 images were collected for each tumour and the FIPS data were averaged to allow comparison of perfusion changes between various tumour regions.

Differences in staining profiles were quantified using a multi-step algorithm to facilitate statistical analysis. Briefly, when the fluorescence intensity of either stain exceeded background, the relative intensity of the carbocyanine staining was compared with the Hoechst staining. Variation by less than a factor of 2 was defined as a '0% change', a 2–3-fold increase in $DiOC_7$ staining relative to Hoechst staining was defined as a '+100% change', etc. Similarly, decreased relative carbocyanine intensities were expressed as negative percentage changes. Percentage changes exceeding ±300% (i.e. > 4-fold changes in relative $DiOC_7$ intensity) roughly corresponded to our previous visual criteria for stain mismatch (Trotter et al, 1989a, 1989b).

### Fluorescence-activated cell sorting and in vivo–in vitro cloning assay

Drug-induced alterations in tumour radiosensitivity were assessed by an in vivo–in vitro cloning assay. Fluorescence-activated cell sorting as part of a cloning assay provides information on the radiosensitivity of various subpopulations of cells within a tumour (Durand, 1994). After X-irradiation, tumour cells can be sorted based on the cellular content of an intravenously injected perfusion stain. By subsequently plating the resultant cell fractions in a cloning assay (Chaplin et al, 1985; Olive et al, 1985; Durand, 1986), the radiosensitivities of various tumour cell subpopulations can be determined. Thus rather than obtaining a net radiosensitivity measurement for an entire tumour from an in vivo–in vitro cloning assay, sorting the tumour cells prior to plating provides information regarding the responses of specific tumour cell subpopulations to a given radiotherapy adjuvant.

Briefly, the animals were treated with the appropriate drug at varying times before whole body 250 keV X-irradiation with a dose rate of 3 Gy $min^{-1}$. Immediately post-irradiation, animals were injected with Hoechst 33342 (1 mg in 0.05 ml PBS) into the lateral tail vein and the tumours were excised 20 min later. This stain concentration was nontoxic to host animals and tumour cells. Excised tumours were washed in ice-cold PBS in order to remove any stain released by the excision process and to inhibit redistribution of the Hoechst. The tumour was finely minced before agitation in an enzyme suspension of 0.5% trypsin and 0.08% collagenase at 37˚C for 40 min; 0.06% DNAse was then added. The cell suspension was gently vortexed, filtered through a 30 μm nylon mesh to remove clumps, and the monodispersed cells were washed and processed through a FACS 440 (Becton Dickinson, Mountain View, California) flow cytometer. Cells were defined on the basis of forward scatter (cell size) and sort windows were automatically set to subdivide the cell population into 8 fractions of differing intracellular Hoechst concentrations (Durand, 1986). The primary assumption of the method is that the Hoechst staining profile of a tumour simulates the oxygenation profile during irradiation. This assumption is valid provided that the time between irradiation and Hoechst injection is short, and that any significant changes in tumour perfusion are sufficiently slow so as not to occur between tumour irradiation and stain injection.

Predetermined numbers of cells were sorted into test tubes containing culture medium. The tubes were then poured and rinsed into 100 mm tissue culture dishes and incubated in 94% air plus 6% $CO_2$ for 2 weeks to allow colony formation. All in vitro techniques used minimal essential medium containing 10% fetal bovine serum and antibiotics. No special additives were used for tumour cell culture, nor were feeder cells, gel cultures, or low oxygen tensions found to improve cell growth or viability of these cell lines (note that these human tumour cell lines were initially selected in tissue culture). In all clonogenicity data presented, we have plotted the ratio of observed colonies to cells plated without correcting for control plating efficiencies (which were in the 20–30% range).

### Statistics

Statistical tests were conducted using SPSS software (SPSS Inc., Chicago, Illinois). 2-sample student's *t*-tests were used to analyse the dual-staining mismatch and radiosensitivity data.

### RESULTS

### Alterations in net tumour perfusion

The modified $^{86}$Rb extraction technique was used to determine the doses of each drug that would yield observable increases in net tumour blood flow. Diltiazem did not significantly increase net perfusion in SiHa or WiDr tumours at doses between 2 and 20 mg $kg^{-1}$ (data not shown). The largest (non-significant) increases in net tumour perfusion for SiHa and WiDr tumours were at diltiazem doses of 5 mg $kg^{-1}$ and 2 mg $kg^{-1}$ respectively. Pentoxifylline increased net tumour perfusion in SiHa tumours at a dose of 5 mg $kg^{-1}$, though the increase was not statistically significant (Figure 3A). A decrease in SiHa net tumour blood flow was also observed 15 minutes after 20 mg $kg^{-1}$ pentoxifylline, but the decrease was not significant and did

© 2001 Cancer Research Campaign 

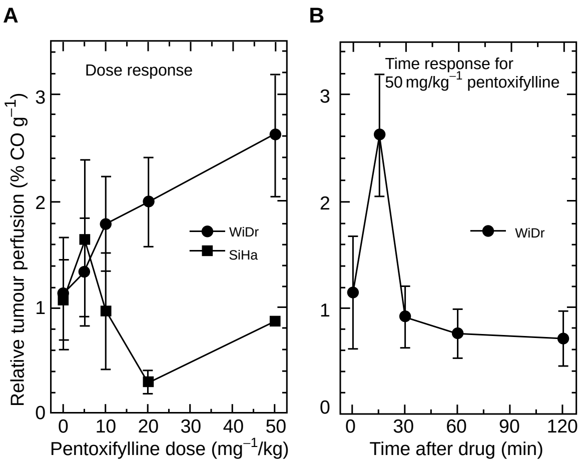

**Figure 3** Relative net tumour perfusion measured by the modified $^{86}$Rb extraction method. (**A**) Relative net tumour perfusion measured 15 minutes after administration of various doses of pentoxifylline (PENTO). SiHa tumours did not demonstrate a significant increase in net perfusion with doses of PENTO between 5 and 50 mg kg$^{-1}$. A decrease in net SiHa tumour perfusion was observed at 20 mg kg$^{-1}$ PENTO, but the decrease was not associated with a concomitant alteration in tumour cell radiosensitivity (see text). WiDr tumours exhibited an approximately linear dose response to PENTO with a maximal observed increase in net tumour perfusion of 130% ± 40% (mean ± SEM) with a dose of 50 mg kg$^{-1}$. *n* = 3–5 animals per data point. (**B**) Relative net perfusion of WiDr tumours measured at various times after administration of 50 mg kg$^{-1}$ pentoxifylline. The increase in net WiDr perfusion observed at 15 minutes returned to control values by 30 minutes after drug dosing. *n* = 3–5 animals per data point not yield an observable effect on SiHa tumour radiosensitivity (data not shown). Net tumour perfusion was increased in WiDr tumours by 130±40% (mean ± SEM) 15 minutes after administration of 50 mg kg$^{-1}$ pentoxifylline (Figure 3A).

The above drug concentrations were used in order to determine the time after drug administration that would yield the greatest increase in net tumour perfusion. Diltiazem did not significantly improve net tumour perfusion at 5 mg kg$^{-1}$ in SiHa tumours or 2 mg kg$^{-1}$ in WiDr tumours at 15, 30, 60 or 120 minutes after drug administration (data not shown). The pentoxifylline-induced increases in net tumour perfusion observed 15 min after drug administration were short-lived in both SiHa and WiDr tumours. Specifically, for WiDr tumours the net tumour perfusion 30 min after drug administration was not significantly different from control levels (Figure 3B).

### Alterations in the micro-regional distribution of tumour blood flow

Micro-regional changes in tumour blood flow were studied 15 and 30 min after administration of pentoxifylline. The distribution of SiHa tumour perfusion was not significantly affected by administration of 5 mg kg$^{-1}$ pentoxifylline (Figure 4A). Subtle redistributions of WiDr tumour perfusion were observed 15 and 30 minutes after 50 mg kg$^{-1}$ pentoxifylline administration when compared to tumours that were not treated with drug between stain injections (Figure 4B). In the WiDr tumours, there was a general decrease in the percentage of vessels that showed reduced perfusion and an increase in the percentage of vessels that showed

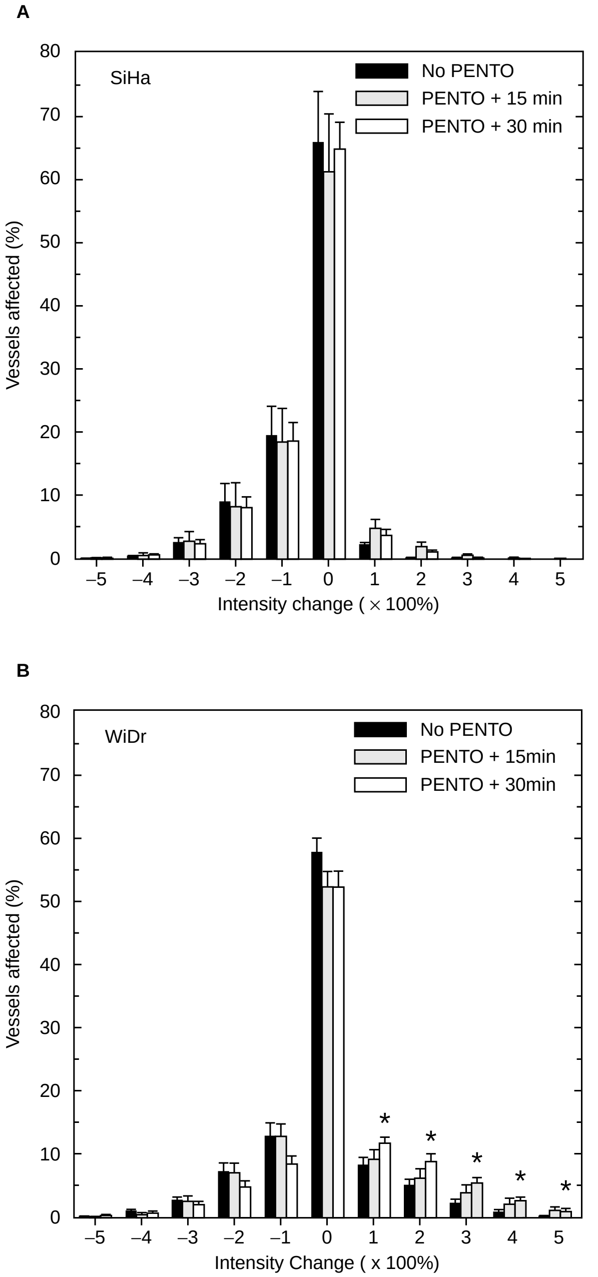

**Figure 4** Dual-staining mismatch data showing alterations in the micro-regional distribution of tumour perfusion 15 and 30 minutes after administration of pentoxifylline (PENTO). (**A**) SiHa tumours did not demonstrate statistically significant alterations in micro-regional perfusion after administration of 5 mg kg$^{-1}$ pentoxifylline. *n* = 6 animals per graph. (**B**) WiDr tumours demonstrated statistically significant increases in micro-regional tumour perfusion 30 minutes after administration of 50 mg kg$^{-1}$ pentoxifylline. *n* = 8–10 animals per graph (* $P \leq 0.05$)

increased perfusion between stain injections. The increases in microregional perfusion 30 minutes after drug administration were statistically significant ($P \leq 0.05$). There was also a decrease in the percentage of vessels that exhibited less than 2-fold changes in perfusion. These data suggest that the pentoxifylline increased blood flow through tumour vessels that would not normally exhibit changes in perfusion over the measurement interval.

 © 2001 Cancer Research Campaign

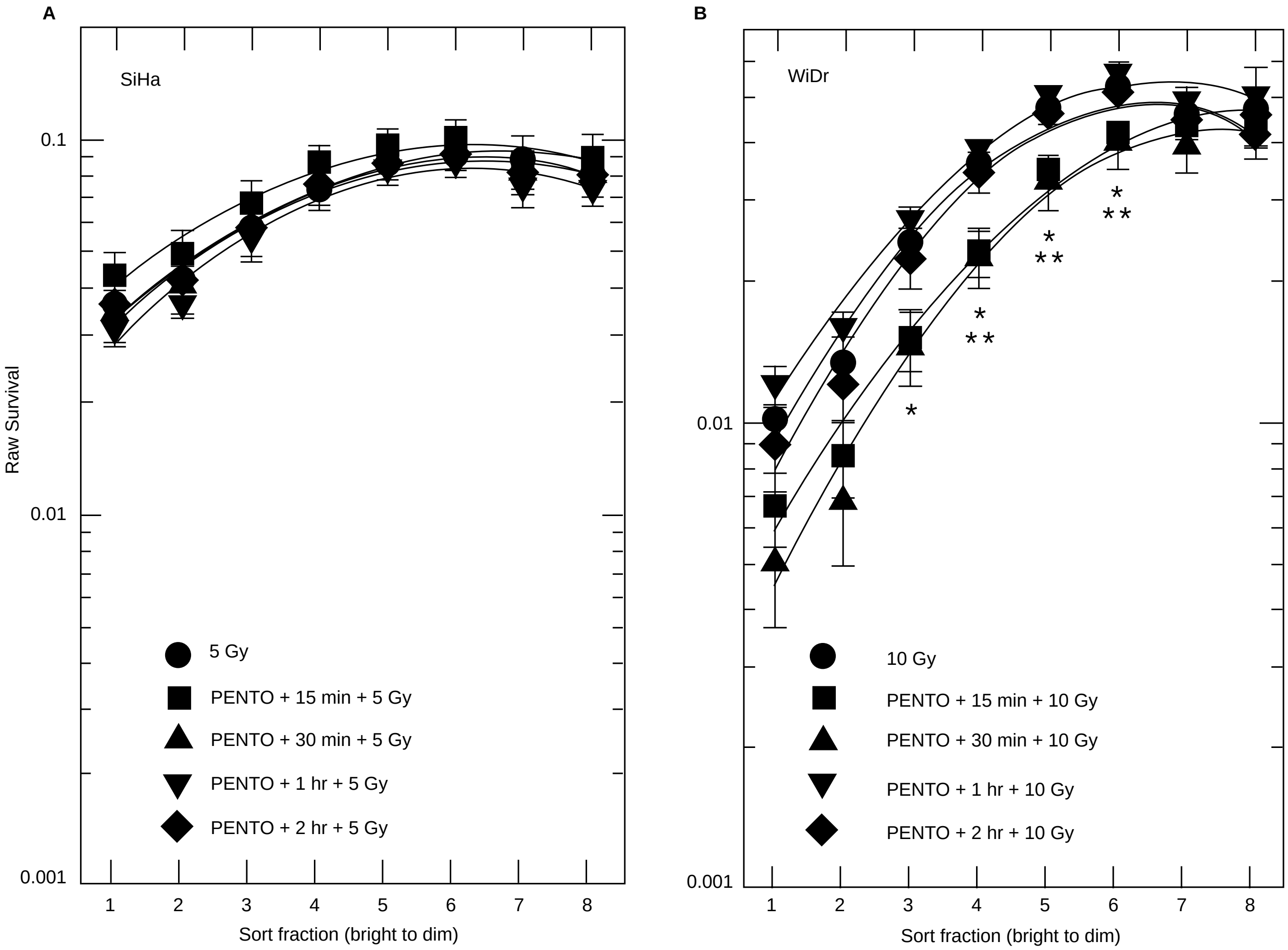

**Figure 5** In vivo–in vitro cloning assay data for SiHa and WiDr tumours with pentoxifylline (PENTO) administered at various times prior to tumour irradiation. Sort fractions were determined based on cellular content of the perfusion stain Hoechst 33342 (see text). (**A**) There was no observable change in SiHa tumour cell radiosensitivity when 5 mg $kg^{-1}$ PENTO was administered at any time prior to tumour irradiation. *n* = 4–6 animals per survival curve. (**B**) WiDr tumour cell radiosensitivity was altered when 50 mg $kg^{-1}$ pentoxifylline was administered at various times prior to tumour irradiation. There were statistically significant increases in the radiosensitivities of sort fractions 3–6 when PENTO was administered 15 minutes prior to irradiation (**P* ≤ 0.05) and in sort fractions 4–6 when PENTO was administered 30 minutes prior to irradiation (***P* ≤ 0.05). *n* = 4–8 animals per survival curve

### Alterations in tumour radiosensitivity

For the cloning assays, the most brightly staining tumour cells (fraction 1) were proximal to functional vasculature and thus represented the cells that were most aerobic during irradiation. Similarly, the most dimly staining tumour cells (fraction 8) were distant from functional vasculature in the tumour and represented the cells that were the least oxygenated. Since we generally consider 2 principal mechanisms of hypoxia generation in solid tumours, the intermediate sort fractions would contain cells that arose from both chronically and transiently hypoxic conditions. The intermediate sort fractions typically represent those cells that existed at static levels of intermediate oxygenation, receiving intermediate levels of stain due to their distance from functional vasculature. In tumours with significant amounts of transient hypoxia however, such as WiDr tumours, intermediate sort fractions can also represent cells that changed oxygenation level during the lifetime of the Hoechst in the circulation ($T_{1/2}$ for Hoechst in murine circulation is 110 seconds (Olive et al, 1985)). With extreme changes in blood vessel perfusion during the circulation lifetime of the Hoechst stain, transiently hypoxic cells may also be present in the most brightly and most dimly staining cell fractions.

SiHa tumour-bearing mice and WiDr tumour-bearing mice were given 5 mg $kg^{-1}$ and 2 mg $kg^{-1}$ diltiazem at various times prior to single irradiation doses of 5 Gy and 10 Gy, respectively. All drug concentrations were chosen based on the maximal increases in net tumour perfusion observed by $^{86}$Rb extraction. As would be expected from the net perfusion data, there were no significant increases in tumour cell radiosensitivity when diltiazem was administered at these doses 15 min to 2 h prior to irradiation of either tumour type (data not shown).

SiHa tumour-bearing mice were given 5 mg $kg^{-1}$ pentoxifylline at various times prior to a single irradiation dose of 5 Gy. The in vivo–in vitro cloning assay data did not indicate any significant changes in tumour cell radiosensitivity between 15 min and 2 h after drug treatment when compared to control mice that received radiation alone (Figure 5A).

WiDr tumour-bearing mice were given 50 mg $kg^{-1}$ pentoxifylline at various times prior to a single irradiation dose of 10 Gy. The in vivo–in vitro cloning assay data indicated increases in the radiosensitivity of tumour cell subfractions 15 and 30 min after

*© 2001 Cancer Research Campaign*

drug administration (Figure 5B). Specifically, the cells that corresponded to intermediate levels of oxygenation exhibited statistically significant increases in radiosensitivity with pentoxifylline treatment prior to irradiation ($P \leq 0.05$). A less marked, non-statistically significant increase in tumour cell radiosensitivity was also observed in the most brightly staining tumour cells. No significant increase in radiosensitivity was observed in the dimmest staining fractions of tumour cells, indicating that the pentoxifylline had no observable radiosensitizing effect on the diffusion-limited hypoxic cells. Since the chronically hypoxic WiDr tumour cells were not affected by the pentoxifylline, the intermediately staining cells that demonstrated pentoxifylline-induced increases in radiosensitivity were most likely transiently hypoxic cells.

### DISCUSSION

Many radiosensitizing drugs have been studied in pre-clinical laboratories as potential adjuvants to radiotherapy. However, there is a limitation to the use of certain agents in that the efficacy of some drugs (as with many chemotherapy agents used to treat primary tumour masses) can be limited by the delivery of the active agent to poorly vascularized tumour regions. Thus an important advantage that drugs such as diltiazem and pentoxifylline have over various other radiosensitizing agents is that the activities of the drugs are not limited by delivery to poorly perfused regions of a tumour mass.

When interpreting the effects of diltiazem or pentoxifylline on the perfusion of experimental tumours, one must consider the tumour system in which the measurements have been performed. Xenografts that have been derived from different cell lines can have very different characteristics in terms of their hypoxic fraction and intrinsic radiosensitivity. In our hands, SiHa tumours demonstrate little evidence of transient changes in perfusion while WiDr tumours are known to contain a relatively large hypoxic fraction consisting of both chronically and transiently hypoxic cells. In addition, sections of SiHa tumours examined microscopically contain larger diameter blood vessels on average than WiDr tumours (data not shown). Thus the narrower vasculature, coupled with the greater hypoxic fraction, in WiDr tumours would be expected to promote a higher level of perfusion-limited hypoxia when compared to SiHa tumours. The intrinsic differences between the hypoxic content of SiHa and WiDr tumours allows each to be used to assess the role of chronic versus acute hypoxia in experimental tumour systems.

Diltiazem did not induce any significant changes in net perfusion of SiHa or WiDr tumours from 15 min to 2 h after administration (data not shown). Our results are in contrast to other data (Wood and Hirst, 1989; Muruganandham et al, 1999) which suggest net tumour perfusion increases at doses between 2 mg $kg^{-1}$ and 100 mg $kg^{-1}$ in other tumour systems. As would be expected from our net perfusion data, there were no significant increases in tumour cell radiosensitivity when diltiazem was administered 15 min to 2 h prior to tumour irradiation (data not shown).

Given the accepted mechanism for diltiazem-induced increases in tumour perfusion (i.e. by affecting systemic arteries), there was no a priori expectation of differential perfusion effects on SiHa versus WiDr tumours with diltiazem administration. However, when considering the mechanism of pentoxifylline-induced changes in tumour perfusion (i.e. by affecting blood cell deformability and thereby allowing blood flow through narrower vasculature), any differences in the functionality of tumour blood vessels could impact potential changes in perfusion. Thus the observation that WiDr tumours have more tortuous vasculature, and hence more transient hypoxia, than SiHa tumours leads to an expectation of dissimilar responses of the 2 tumours to pentoxifylline.

Pentoxifylline non-significantly increased the net perfusion of SiHa tumours 15 min after a dose of 5 mg $kg^{-1}$ (Figure 3A) and there were no observed increases in net tumour perfusion from 30 min to 2 h after drug administration (data not shown). There were also no observable increases in the radiosensitivity of any SiHa tumour cell subpopulations when pentoxifylline was administered prior to tumour irradiation (Figure 5A). Since SiHa tumours contain relatively little transient hypoxia, the observation that pentoxifylline did not influence SiHa radiosensitivity suggests the drug did not increase the oxygenation level of chronically hypoxic SiHa tumour cells.

Pentoxifylline increased the net perfusion of WiDr tumours by 130 ± 40% (mean ± SEM) 15 min after administration of a dose of 50 mg $kg^{-1}$ (Figure 3A), and the net tumour blood flow returned to control levels by 30 minutes (Figure 3B). The effective pentoxifylline dose of 50 mg $kg^{-1}$ agrees with other published data (Song et al, 1992, 1994; Honess et al, 1993; Kelleher et al, 1998), though the maximal values of increased tumour perfusion varies among the tumour systems. The observed increase in net tumour perfusion after 15 minutes correlated with an increase in blood flow through partially occluded tumour blood vessels as suggested by the dual-staining mismatch data (Figure 4B). Interestingly, the redistribution of tumour perfusion on a micro-regional level was found to be at least 30 min in duration even though there was no observable increase in net tumour perfusion at that time. These data suggest that micro-regional redistributions of tumour blood flow may not necessarily be associated with concomitant changes in net tumour perfusion, and thus may not be detectable via the $^{86}Rb$ extraction method. Based on the mismatch data, an increase in the oxygenation and radiosensitivity of normally perfusion-limited hypoxic cells would be expected up to 30 minutes after pentoxifylline administration.

The in vivo–in vitro cloning assay data showed increases in WiDr tumour cell radiosensitivity when pentoxifylline was given 15 and 30 min prior to tumour irradiation (Figure 5B). When considering the effect of pentoxifylline on the radiosensitivity of different WiDr tumour cell subpopulations, the sort fractions that corresponded to tumour cells at intermediate levels of oxygenation exhibited statistically significant increases in radiosensitivity ($P \leq 0.05$). When taken with the dual staining mismatch data 30 minutes after drug administration, the pentoxifylline-induced increase in the radiation response of intermediately staining tumour cells was likely due to reoxygenation of perfusion-limited hypoxic cells. The increase in WiDr radiosensitivity after 30 min was of comparable magnitude to the increased radiosensitivity associated with the net perfusion increase after 15 minutes. These data suggest that the redistribution of tumour blood flow to increase oxygen delivery to intermediately oxygenated cells is at least as important as increasing net tumour blood flow in terms of increasing the radiosensitivity of WiDr tumours.

Determining the identity of cells that respond to a particular therapeutic intervention has several implications for clinical radiotherapy. For example, treatments designed to increase the

*© 2001 Cancer Research Campaign*

net oxygen content of the blood during radiotherapy would be expected to have a radiosensitization effect on chronically hypoxic cells, but would not likely affect the response of cells made transiently hypoxic by temporary fluctuations in tumour perfusion (Chaplin et al, 1986). However, before strategies for targeting specific cell subpopulations can be transferred to the clinical situation, further characterization of tumour hypoxia is necessary. Specifically, data regarding the presence of transient hypoxia in clinical tumours has been anecdotal thus far, and further studies are necessary to determine if transient hypoxia is present in sufficient quantity to impact clinical tumour response (Durand and Aquino-Parsons, 2001).

The challenge of clinically targeting specific tumour cell subpopulations is exacerbated by current definitions of tumour response to therapy. In the clinical situation, tumours are generally defined as ‘responsive’ to therapy when tumour shrinkage occurs. Thus in order to observe a ‘response’ to therapy, a majority of tumour cells must both die and disappear from the tumour bulk during treatment. However, when considering the goal of tumour ‘cure’, kinetics studies in experimental tumours have shown that it is essential that the minority of maximally resistant tumour cells are also destroyed during treatment (Durand, 1993, 1994). The difficulty inherent in defining a radiotherapy intervention that affects a minority of tumour cells is evident in the clinical situation so long as the majority of tumour cells dictate clinical tumour ‘response’. Thus the further refinement of pre-clinical and clinical laboratory techniques designed to elucidate the responses of specific tumour cell subpopulations to various therapeutic interventions is warranted.

As suggested in a recent paper by Wouters and Brown (1997), cells at intermediate levels of oxygenation may have a substantial effect on the overall response of a tumour to radiation. Our data support this assertion in that drug-induced redistributions of tumour blood flow to favour increased oxygen delivery to intermediately oxygenated cells yielded increases in tumour cell response to a large, single dose of radiation. However, the response of tumours to multiple fractions of radiation administered in clinical radiotherapy protocols may also be influenced by the presence of transiently hypoxic cells. Further studies are necessary in order to assess the presence and potential impact that transiently hypoxic cells may have on tumour response to therapy.

### ACKNOWLEDGEMENTS

Technical assistance from Denise McDougal and Darrell Trendall is gratefully acknowledged. This work was supported by the National Cancer Institute of Canada with funds from the Canadian Cancer Society.

### REFERENCES

Arcuri LB, Fredrickson MK and Ziegler KM (1998a) Diltiazem Hydrochloride. In: *AHFS Drug Information*, McEvoy GK (ed) pp 1317–1324. American Society of Health-System Pharmacists: Bethesda

Arcuri LB, Fredrickson MK and Ziegler KM (1998b) Pentoxifylline. In: *AHFS Drug Information*, McEvoy GK (ed) pp 1238–1242. American Society of Health-System Pharmacists: Bethesda

Armstrong Jr M, Needham D, Hatchell DL and Nunn RS (1990) Effect of pentoxifylline on the flow of polymorphonuclear leukocytes through a model capillary. *Angiology* **41**: 253–262

Chaplin DJ, Durand RE and Olive PL (1985) Cell selection from a murine tumor using the fluorescent probe Hoechst 33342. *Br J Cancer* **51**: 569–572

Chaplin DJ, Durand RE and Olive PL (1986) Acute hypoxia in tumors: implications for modifiers of radiation effects. *Int J Radiat Oncol Biol Phys* **12**: 1279–1282

Durand RE (1986) Use of a cell sorter for assays of cell clonogenicity. *Cancer Res* **46**: 2775–2778

Durand RE (1993) Cell kinetics and repopulation during multifraction irradiation of spheroids: implications for clinical radiotherapy. *Sem Radiat Oncol* **3**: 105–114

Durand RE (1994) The influence of microenvironmental factors during cancer therapy. *in vivo* **8**: 691–702

Durand RE and LePard NE (1994) Modulation of tumor hypoxia by conventional chemotherapeutic agents. *Int J Radiat Oncol Biol Phys* **29**: 481–486

Durand RE and LePard NE (1995) Contribution of transient blood flow to tumour hypoxia in mice. *Acta Oncol* **34**: 317–324

Durand RE and Aquino-Parsons C (2001) Intermittent tumour blood flow: implications for therapy. *Acta Oncologica* (in press)

Ehrly AM (1978) The effect of pentoxifylline on the flow properties of human blood. *Curr Med Res Opin* **5**: 608–613

Friedl F, Kimura I, Osato T and Ito Y (1970) Studies on a new human cell line (SiHa) derived from carcinoma of uterus. I. Its establishment and morphology. *Proc Soc Exp Biol Med* **135**: 543–545

Greenberg DA (1987) Calcium channels and calcium antagonists. *Ann Neurol* **21**: 317–330

Gullino PM and Grantham FH (1961) Studies on the exchange of fluids between host and tumor. II. The blood flow of hepatomas and other tumors in rats and mice. *J Natl Cancer Inst* **27**: 1465–1491

Hakim TS and Macek AS (1988) Effect of hypoxia on erythrocyte deformability in different species. *Biorheology* **25**: 857–868

Honess DJ, Dennis IF and Bleehen NM (1993) Pentoxifylline: Its pharmacokinetics and ability to improve tumour perfusion and radiosensitivity in mice. *Radiother Oncol* **28**: 208–218

Honess DJ, Andrews MS, Ward R and Bleehen NM (1995) Pentoxifylline increases RIF-1 tumour $pO_2$ in a manner compatible with its ability to increase relative tumour perfusion. *Acta Oncol* **34**: 385–389

Kelleher DK, Thews O and Vaupel P (1998) Regional perfusion and oxygenation of tumors upon methylxanthine derivative administration. *Int J Radiat Oncol Biol Phys* **42**: 861–864

Lee I, Kim JH, Levitt SH and Song CW (1992) Increases in tumor response by pentoxifylline alone or in combination with nicotinamide. *Int J Radiat Oncol Biol Phys* **22**: 425–429

Lee I, Levitt SH and Song CW (1993) Improved tumour oxygenation and radiosensitization by combination with nicotinamide and pentoxifylline. *Int J Radiat Biol* **64**: 237–244

Lee I, Boucher Y, Demhartner TJ and Jain RK (1994) Changes in tumour blood flow, oxygenation and interstitial fluid pressure induced by pentoxifylline. *Br J Cancer* **69**: 492–496

Muruganandham M, Kasiviswanathan A, Jagannathan NR, Raghunathan P, Jain PC and Jain V (1999) Diltiazem enhances tumor blood flow: MRI study in a murine tumor. *Int J Radiat Oncol Biol Phys* **43**: 413–421

Noguchi P, Wallace R, Johnson J, Earley EM, O’Brien S, Ferrone S, Pellegrino MA, Mustein J, Needy C, Browne W and Petricciani J (1979) Characterization of the WIDR: a human colon carcinoma cell line. *In Vitro* **15**: 401–408

Olive PL, Chaplin DJ and Durand RE (1985) Pharmacokinetics, binding and distribution of Hoechst 33342 in spheroids and murine tumours. *Br J Cancer* **52**: 739–746

Sapirstein LA (1958) Regional blood flow by fractional distribution of indicators. *Am J Physiol* **193**: 161–168

Song CW, Hasegawa T, Kwon HC, Lyons JC and Levitt SH (1992) Increase in tumor oxygenation and radiosensitivity caused by pentoxifylline. *Radiat Res* **130**: 205–210

Song CW, Makepeace CM, Griffin RJ, Hasegawa T, Osborn JL, Choi I-B and Nah BS (1994) Increase in tumor blood flow by pentoxifylline. *Int J Radiat Oncol Biol Phys* **29**: 433–437

Thomlinson RH and Gray LH (1955) The histological structure of some human lung cancers and the possible implications for radiotherapy. *Br J Cancer* **9**: 539–549

Trotter MJ, Chaplin DJ, Durand RE and Olive PL (1989*a*) The use of fluorescent probes to identify regions of transient perfusion in murine tumors. *Int J Radiat Oncol Biol Phys* **16**: 931–934

Trotter MJ, Chaplin DJ and Olive PL (1989*b*) Use of a carbocyanine dye as a marker of functional vasculature in murine tumours. *Br J Cancer* **59**: 706–709

UKCCR (1998) United Kingdom co-ordinating committee on cancer research (UKCCR) guidelines for the welfare of animals in experimental neoplasia (second edition). *Br J Cancer* **77**: 1–10

© 2001 Cancer Research Campaign 

Van Nueten JM and Vanhoutte PM (1980) Improvement of tissue perfusion with inhibitors of calcium ion influx. *Biochem Pharm* **29**: 479–481
Wood PJ and Hirst DG (1989) Modification of tumour response by calcium antagonists in the SCCVII/St tumour implanted at two different sites. *Int J Radiat Biol* **56**: 355–367
Wouters BG and Brown JM (1997) Cells at intermediate oxygen levels can be more important than the "hypoxic fraction" in determining tumor response to fractionated radiotherapy. *Radiat Res* **147**: 541–550
Zanelli GD and Fowler JF (1974) The measurement of blood perfusion in experimental tumors by uptake of $^{86}$Rb. *Cancer Res* **34**: 1451–1456

 *© 2001 Cancer Research Campaign*

## References

[PDF_00749] Armstrong M., Needham D., Hatchell D. L., Nunn R. S. (1990). Effect of pentoxifylline on the flow of polymorphonuclear leukocytes through a model capillary.. Angiology.

[PDF_00754] Chaplin D. J., Durand R. E., Olive P. L. (1986). Acute hypoxia in tumors: implications for modifiers of radiation effects.. Int J Radiat Oncol Biol Phys.

[PDF_00752] Chaplin D. J., Durand R. E., Olive P. L. (1985). Cell selection from a murine tumour using the fluorescent probe Hoechst 33342.. Br J Cancer.

[PDF_00766] Durand R. E., LePard N. E. (1995). Contribution of transient blood flow to tumour hypoxia in mice.. Acta Oncol.

[PDF_00762] Durand R. E., LePard N. E. (1994). Modulation of tumor hypoxia by conventional chemotherapeutic agents.. Int J Radiat Oncol Biol Phys.

[PDF_00757] Durand R. E. (1986). Use of a cell sorter for assays of cell clonogenicity.. Cancer Res.

[PDF_00759] Durand RE (1993). Cell Kinetics and Repopulation During Multifraction Irradiation of Spheroids: Implications for Clinical Radiotherapy.. Semin Radiat Oncol.

[PDF_00772] Friedl F., Kimura I., Osato T., Ito Y. (1970). Studies on a new human cell line (SiHa) derived from carcinoma of uterus. I. Its establishment and morphology.. Proc Soc Exp Biol Med.

[PDF_00777] GULLINO P. M., GRANTHAM F. H. (1961). Studies on the exchange of fluids between host and tumor. II. The blood flow of hepatomas and other tumors in rats and mice.. J Natl Cancer Inst.

[PDF_00775] Greenberg D. A. (1987). Calcium channels and calcium channel antagonists.. Ann Neurol.

[PDF_00780] Hakim T. S., Macek A. S. (1988). Effect of hypoxia on erythrocyte deformability in different species.. Biorheology.

[PDF_00785] Honess D. J., Andrews M. S., Ward R., Bleehen N. M. (1995). Pentoxifylline increases RIF-1 tumour pO2 in a manner compatible with its ability to increase relative tumour perfusion.. Acta Oncol.

[PDF_00782] Honess D. J., Dennis I. F., Bleehen N. M. (1993). Pentoxifylline: its pharmacokinetics and ability to improve tumour perfusion and radiosensitivity in mice.. Radiother Oncol.

[PDF_00789] Kelleher D. K., Thews O., Vaupel P. (1998). Regional perfusion and oxygenation of tumors upon methylxanthine derivative administration.. Int J Radiat Oncol Biol Phys.

[PDF_00798] Lee I., Boucher Y., Demhartner T. J., Jain R. K. (1994). Changes in tumour blood flow, oxygenation and interstitial fluid pressure induced by pentoxifylline.. Br J Cancer.

[PDF_00792] Lee I., Kim J. H., Levitt S. H., Song C. W. (1992). Increases in tumor response by pentoxifylline alone or in combination with nicotinamide.. Int J Radiat Oncol Biol Phys.

[PDF_00795] Lee I., Levitt S. H., Song C. W. (1993). Improved tumour oxygenation and radiosensitization by combination with nicotinamide and pentoxifylline.. Int J Radiat Biol.

[PDF_00801] Muruganandham M., Kasiviswanathan A., Jagannathan N. R., Raghunathan P., Jain P. C., Jain V. (1999). Diltiazem enhances tumor blood flow: MRI study in a murine tumor.. Int J Radiat Oncol Biol Phys.

[PDF_00804] Noguchi P., Wallace R., Johnson J., Earley E. M., O'Brien S., Ferrone S., Pellegrino M. A., Milstien J., Needy C., Browne W. (1979). Characterization of the WIDR: a human colon carcinoma cell line.. In Vitro.

[PDF_00807] Olive P. L., Chaplin D. J., Durand R. E. (1985). Pharmacokinetics, binding and distribution of Hoechst 33342 in spheroids and murine tumours.. Br J Cancer.

[PDF_00810] SAPIRSTEIN L. A. (1958). Regional blood flow by fractional distribution of indicators.. Am J Physiol.

[PDF_00812] Song C. W., Hasegawa T., Kwon H. C., Lyons J. C., Levitt S. H. (1992). Increase in tumor oxygenation and radiosensitivity caused by pentoxifylline.. Radiat Res.

[PDF_00815] Song C. W., Makepeace C. M., Griffin R. J., Hasegawa T., Osborn J. L., Choi I. B., Nah B. S. (1994). Increase in tumor blood flow by pentoxifylline.. Int J Radiat Oncol Biol Phys.

[PDF_00818] THOMLINSON R. H., GRAY L. H. (1955). The histological structure of some human lung cancers and the possible implications for radiotherapy.. Br J Cancer.

[PDF_00821] Trotter M. J., Chaplin D. J., Durand R. E., Olive P. L. (1989). The use of fluorescent probes to identify regions of transient perfusion in murine tumors.. Int J Radiat Oncol Biol Phys.

[PDF_00824] Trotter M. J., Chaplin D. J., Olive P. L. (1989). Use of a carbocyanine dye as a marker of functional vasculature in murine tumours.. Br J Cancer.

[PDF_00829] Van Nueten J. M., Vanhoutte P. M. (1980). Improvement of tissue perfusion with inhibitors of calcium ion influx.. Biochem Pharmacol.

[PDF_00831] Wood P. J., Hirst D. G. (1989). Modification of tumour response by calcium antagonists in the SCVII/St tumour implanted at two different sites.. Int J Radiat Biol.

[PDF_00834] Wouters B. G., Brown J. M. (1997). Cells at intermediate oxygen levels can be more important than the "hypoxic fraction" in determining tumor response to fractionated radiotherapy.. Radiat Res.

[PDF_00837] Zanelli G. D., Fowler J. F. (1974). The measurement of blood perfusion in experimental tumors by uptake of 86Rb.. Cancer Res.

